# Syndrome thoracique aigu chez une gestante AS méconnue: à propos d’un cas

**DOI:** 10.11604/pamj.2019.34.110.19001

**Published:** 2019-10-24

**Authors:** Yvette Neema UfoyMungu, Jean Didier Bosenge Nguma, Gédéon Katenga Bosunga, Roland Marini Djangeing’a, Salomon Batina Agasa

**Affiliations:** 1 Département de Gynécologie-Obstétrique, Cliniques Universitaires de Kisangani, Université de Kisangani, Kisangani, République Démocratique du Congo; 2 Département de Pharmacie, Faculté de Médecine et de Pharmacie, Université de Kisangani, Kisangani, République Démocratique du Congo; 3 Laboratoire de Chimie Analytique Pharmaceutique, Département de Pharmacie, Université de Liège, Belgique; 4 Département de Médecine Interne, Cliniques Universitaires de Kisangani, Université de Kisangani, Kisangani, République Démocratique du Congo

**Keywords:** Trait drépanocytaire, syndrome thoracique aigu, grossesse, Kisangani, Sickle cell trait, acute thoracic syndrome, pregnancy, Kisangani

## Abstract

Nous rapportons un cas de syndrome thoracique aigu (STA) diagnostiqué chez une gestante congolaise dépistée trait drépanocytaire à l'occasion de son hospitalisation pour fièvre et symptômes respiratoires. L'ensemble d'explorations paracliniques à visée étiologique réalisées était non contributif, la radiographie du thorax a mis en évidence des infiltrats à la base des poumons. Les dépistages au kit de dépistage Sickle scan test confirmé au moyen de la chromatographie liquide couplée à la spectrométrie de masse ont révélé que la gestante est porteuse du trait drépanocytaire AS. Les symptômes observés étaient quasi réfractaires au traitement instauré. Une évolution favorable inattendue a été observée après l'expulsion fœtale. Les cliniciens devraient considérer le STA comme un diagnostic envisageable chez les traits drépanocytaires AS et dont l'évolution chez une gestante peut être favorable en postpartum.

## Abstract (en)

We report a case of acute thoracic syndrome diagnosed in a Congolese pregnant woman in whom sickle-cell trait was detected after admission to hospital for fever and respiratory symptoms. Paraclinical examinations performed to detect the cause of the disease were unhelpful. Chest X-ray showed infiltrates at the base of the lungs. Sickle SCAN test kit confirmed by liquid chromatography associated with mass spectrometry showed that the patient had sickle cell trait Symptoms were quite refractory to treatment. A favorable outcome was observed after expulsion of the fetus. Clinicians should suspect acute thoracic syndrome in pregnant women with sickle-cell trait because outcome can be favorable in the postpartum period.

## Introduction

La drépanocytose est une maladie génétique autosomique récessive liée à la présence dans l'hématie de l'hémoglobine S (Hb S). La forme homozygote SS est grave et se caractérise par une morbidité et une mortalité élevées [1] en Afrique subsaharienne(ASS). La grossesse chez une patiente drépanocytaire représente une situation à risque tant pour la mère (crises vaso-occlusives, aggravation de l'anémie, syndrome thoracique aigu etc.) que pour le foetus (retard de croissance intra-utérin, prématurité, décès surtout au troisième trimestre) [2]. Les sujets hétérozygotes AS sont asymptomatiques [3]. En ASS, la prévalence élevée des de porteurs de traits drépanocytaires (AS) en fait un problème de santé publique. Elle varie de 10 à 40% en Afrique équatoriale [4] et de 25 à 30% en République Démocratique du Congo (RDC) [5]. Le STA est une des complications pulmonaires, fréquente chez les drépanocytaires homozygotes SS et les hétérozygotes SC [6]. Il survient rarement chez les hétérozygotes AS [3]. Nous présentons le cas d'une découverte fortuite du statut hémoglobinique AS chez une gestante avec STA, réfractaire au traitement, dont l'issue maternelle était favorable en postpartum.

## Patient et observation

Une gestante de 30ans, P_3_ G_6_ A_2_+_1_ était suivie, depuis une semaine dans un centre de santé pour fièvre intermittente et polyarthralgie sur une grossesse de 25 semaines. Elle a bénéficié des injections des antipaludéens, sans succès. Au contraire, à une semaine de son transfert aux Cliniques Universitaires de Kisangani, sa clinique s'était enrichie de douleur thoracique localisée aux bases pulmonaires d'installation progressive, d'intensité croissante et quasi permanente; accompagnée d'une toux grasse avec expectorations striées de sang et de polypnée.

C'est une multipare, multigeste, avec deux notions d'avortements survenus dans un contexte fébrile. De ses 3 enfants, deux sont décédés à l'âge de 5 ans dans un contexte de fièvre et d'anémie. L'enfant en vie est âgé de 1 an et 7 mois. Les grossesses antérieures ont été marquées par des épisodes des douleurs ostéo-articulaires récurrentes et invalidantes prises en charge soit à l'hôpital comme cas de paludisme, soit par les tradipraticiens, avec évolution favorable en postpartum tardif. Les mouvements actifs fœtaux étaient présents et la patiente avait une asthénie physique. Son état général était altéré par une fièvre quantifiée à 39°C, un amaigrissement de 6% et une attitude alitée passive. Les conjonctives palpébrales étaient pâles et les bulbaires sub-ictériques. La gestante pesait 49 kg pour 1,65m de taille. Elle était tachycarde à 112 battements par minute et polypneique à 36 cycles par minute. La palpation réveillait une sensibilité basithoracique bilatérale. Nous avons objectivé une splénomégalie au stade 3 de Hackett.

**Figure 1:**
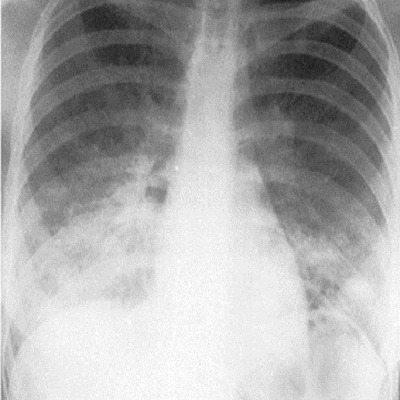
Radiographie du thorax évoquant une infiltration des bases pulmonaires

**Figure 2:**
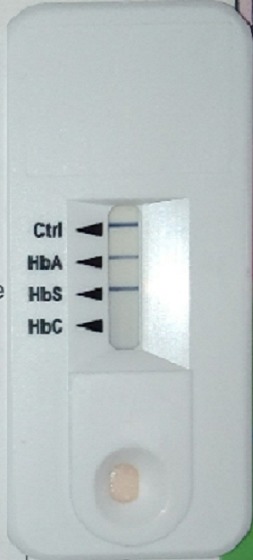
Sickle scan test indiquant le statut hemoglobinique AS

La hauteur utérine était de 23cm, l'utérus non contractile et le reste de l'examen obstétrical était sans particularités. La radiographie du thorax a objectivé des infiltrats diffus aux deux bases pulmonaires (Figure 1); l'hémogramme a révélé une anémie à 5,6gr%, normochrome normocytaire (VGM= 84.2 fl et TCMH = 32.1 pg), LDH à 1789 UI/l, la CRP à 24 mg/l et le sickle scan test a révélé qu'elle était trait drépanocytaire AS (Figure 2), confirmé par la chromatographie liquide couplée à la spectrométrie de masse.

Les hémocultures pendant les pics fébriles, la recherche des agglutinines irrégulières, les tests sérologiques de l'Hépatite B et C, la sérologie VIH, l'examen cytobactériologique des urines, la goutte épaisse, l'examen des crachats à la coloration de Ziehl Nelseen, de même que la recherche du gène X-per étaient tous négatifs. L'électrocardiogramme était normal et l'échographie obstétricale avait révélé une grossesse monofoetale normo-évolutive.

Nous avons suspecté un STA sur grossesse de 25 semaines chez une drépanocytaire hétérozygote. Nous l'avons hospitalisée et mise sous oxygène, ceftriaxone, azythromycine, paracétamol, cardioaspirine, tramadol au besoin et acide folique. Nous avons en outre administré au total 700 ml le culot érythrocytaire, la papavérine et l'avons régulièrement réhydratée. Dans son évolution, il y a eu persistance de la fièvre oscillant entre 38,5 et 40°C et de la douleur thoracique avec des brèves périodes de rémission.

A 26 semaines, l'échographie a objectivé des zones caverneuses coexistant avec une micro calcification marquée du placenta et le foetus de sexe masculin avait un poids estimatif de 1000 grammes. A 27 semaines, elle a accouché prématurément d'un nouveau-né mort, sans malformations congénitales visibles. Le placenta totalement calcifié a pesé 150 grammes. Les symptômes se sont progressivement amendés en postpartum moyen; hormis la fièvre dont la défervescence progressive s'est prolongée en postpartum tardif. Au jour 30 du postpartum, la patiente était afébrile. Elle pesait 50kg, la splénomégalie était au stade 2 de Hackett et l'hémoglobine à 9gr%.

## Discussion

La prévalence des gestantes porteuses du trait drépanocytaire semble inconnue en RDC où le dépistage du statut hémoglobinique n'est pas systémique pendant les consultations prénatales, moins encore au cours des examens prénuptiaux.

Chez notre patiente, le diagnostic du statut hémoglobinique AS était motivé par la présence d'une anémie associée à la poly arthralgie, à la fièvre et aux symptômes respiratoires réfractaires aux traitements; face aux antécédents éloquents. La proportion de l'HB S n'a pas été quantifiée par manque l'électrophorèse capillaire et de la chromatographie liquide à haute performance dans le milieu. Il faut noter la relative rareté des complications maternelles chez la femme AS, mais elles restent néanmoins possibles [7].

S'agissant des complications spécifiques à la grossesse sur trait drépanocytaire, l'accent a été mis sur la bactériurie asymptomatique, la pré éclampsie, la perte des produits de conception après le premier trimestre et les complications cardiovasculaires [4].

En Guadeloupe une étude portant sur 68 gestantes drépanocytaires avait révélé que le STA aigu touchait tous les génotypes avec une plus grande fréquence chez les SS [1]. Le STA est reconnu comme une des complications possibles chez les gestantes avec trait drépanocytaire. Quelques cas ont été rapportés chez les opérés, les diabétiques et les athlètes [8]. Des cas de STA d'issue fatale chez les traits drépanocytaires ont également été signalés notamment le décès maternel [4] ainsi que le décès d'un afro-américain après avoir travaillé dans un grenier en jour d'été [9].

Le STA associe des signes respiratoires à une fièvre qui dépasse rarement 39°C et des images radiologiques récentes. Les recherches bactériologiques sont en règle négatives surtout chez l'adulte [6]. Sa sémiologie clinique n'est pas discriminatoire, il s'accompagne chez 80% de patients d'une crise vaso-occlusive qui la précède dans 50% de cas. Une étiologie n'est retrouvée que dans 30 à 70% de cas en dépit de la complexité des investigations pratiquées [10]. Dans notre cas, le lavage broncho alvéolaire n'a pas été effectué faute d'expertise. Les mécanismes de survenue sont complexes, aussi bien infectieuses que non infectieuses. Cependant, les mécanismes les plus fréquemment évoqués sont la libération d'acides gras toxiques pour la paroi alvéolaire dans l'embolie graisseuse [10] et les atélectasies secondaires aux infarctus costaux. La falciformation des hématies au niveau de la microcirculation pulmonaire est aussi évoquée. Le traitement comprend systématiquement l'administration très rapide des morphiniques, une hyperhydratation, l'oxygénothérapie, l'apport de folates et la correction de l'acidose. L'échange transfusionnel ou la transfusion simple est indispensable pour traiter les formes graves pour ramener le taux d'Hb S inférieur ou égal à 50%. Une antibiothérapie dirigée contre les pneumocoques est justifée, même si l'origine n'est pas infectieuse. Il a été proposé l'administration de monoxyde d'azote (NO) dont l'inhalation améliore le flux sanguin et la saturation de l'hémoglobine en oxygène [6]. Hormis l'inhalation de NO inaccessible dans notre milieu, toutes les mesures thérapeutiques ont été initiées chez notre patiente sans succès.

Le STA est la première cause de décès du drépanocytaire en dehors de la grossesse, mais aussi une des causes les plus fréquentes pendant la grossesse. Le décès survient dans 9 à 25% de cas selon les séries [10]. En postpartum, le décès peut être lié à une embolie pulmonaire ou survenir à la suite d'une complication majeure: complications graves des crises vaso-occlusives dont le STA, infection et crise d'anémie aiguë [6]. Nous pensons que l'accouchement a positivement modifié le cours de la maladie dans notre cas.

## Conclusion

Le dépistage du statut hémoglobinique devrait être systématique chez les gestantes avec anémie, douleurs osteo articulaires; avec antécédent des fausses couches et/ou de grossesses arrêtées. Chez une gestante drépanocytaire, même hétérozygote, la douleur thoracique accompagnée de fièvre doit faire penser avant tout à un syndrome thoracique aigu dont l'évolution peut s'améliorer par l'interruption de la grossesse.

## Conflits d’intérêts

Les auteurs ne déclarent aucun conflit d'intérêts.
